# A cortical attractor network with dynamic synapses

**DOI:** 10.1186/1471-2202-12-S1-P187

**Published:** 2011-07-18

**Authors:** Pradeep Krishnamurthy, Gilad Silberberg, Anders Lansner

**Affiliations:** 1 Department of Numerical analysis and Computer Science, Stockholm University, 114 21 Stockholm, Sweden; 2 Nobel Institute of Neurophysiology, Department of Neuroscience, Karolinska Institute, Stockholm, Sweden; 3 Department of Computational Biology, Royal Institute of Technology (KTH), 114 21 Stockholm, Sweden

## 

**Figure 1:**
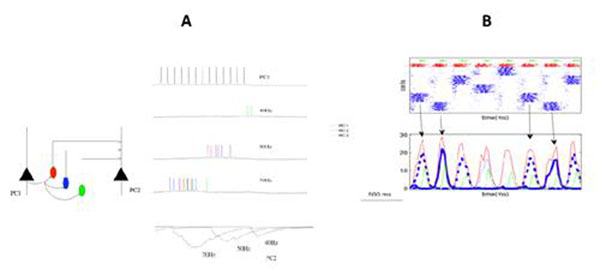
(A) PC-MC microcircuitry showing the disynaptic pathway between the PC mediated by MC showing frequency dependent recuitment and frequency dependent disynaptic inhibition of PC. (B) Raster plot (top) and average firing rate (bottom) of all the cells. The cells are colour-coded: PC (blue), BC (red) and MC (green).

Neocortical inhibitory interneurons play a critical role in shaping the network activity patterns by directly controlling the firing rates of pyramidal cells (PC) [1]. Evidences are accumulating for the possible role of Martinotti cells (MC), which are dendrite-targeting interneurons that receive strongly facilitating synapses from PC, as opposed to basket cells (BC) that are soma targeting and receive strongly depressing synapses [2]. We have previously developed a network model of neocortical layers 2/3 [3] and we here extend this set-up to explore the possible division of labour between basket and Martinotti cells. We used single-compartment cells taken from Pospischill et al. [4] and implemented in NEURON [5]. Short-term depression and facilitation were incorporated for all glutamatergic and GABAergic synapses according to the formalism of Tsodyks & Markram [6] with parameters tuned from traces provided by Silberberg et al. [2]. We commenced with reproducing in our model the PC – MC microcircuit, as previously described by Silberberg & Markram [2], and reproduced (a) frequency dependent disynaptic inhibition of PC and (b) frequency dependent recruitment of MC (Figure 1A). Thereafter, we integrated this microcircuit into our cortical network model to study the effects of MC on the attractor dwell time while the network is spontaneously hopping between the attractor states (stored memories) in the absence of external input. Raster plot and average firing rate (Figure 1B) show that BC that receive depressing synapses has a high firing rate at the beginning of the attractor state which then tapers off. On the other hand, MC that receive facilitating synapses display a late onset of activation and tend to terminate an ongoing attractor state. Cortex is provided with many mechanisms, e.g. spike frequency adaptation, synaptic depression of PC-PC synapses and late firing MC, to control its activity levels and termination of attractors. However, our simulations show that MC inhibition could be a dominating factor, the high divergence of MC to PC connections also assists this.
